# Carbon emissions tax policy of urban road traffic and its application in Panjin, China

**DOI:** 10.1371/journal.pone.0196762

**Published:** 2018-05-08

**Authors:** Longhai Yang, Xiaowei Hu, Lin Fang

**Affiliations:** 1 School of Transportation Science and Engineering, Harbin Institute of Technology, Harbin, Heilongjiang, China; 2 Shanghai Municipal Planning Design Research Institute, Shanghai, China; Beihang University, CHINA

## Abstract

How to effectively solve traffic congestion and transportation pollution in urban development is a main research emphasis for transportation management agencies. A carbon emissions tax can affect travelers’ generalized costs and will lead to changes in passenger demand, mode choice and traffic flow equilibrium in road networks, which are of significance in green travel and low-carbon transportation management. This paper first established a mesoscopic model to calculate the carbon emissions tax and determined the value of this charge in China, which was based on road traffic flow, vehicle speed, and carbon emissions. Referring to existing research results to calibrate the value of time, this paper modified the traveler’s generalized cost function, including the carbon emissions tax, fuel surcharge and travel time cost, which can be used in the travel impedance model with the consideration of the carbon emissions tax. Then, a method for analyzing urban road network traffic flow distribution was put forward, and a joint traffic distribution model was established, which considered the relationship between private cars and taxis. Finally, this paper took the city of Panjin as an example to analyze the road traffic carbon emissions tax’s impact. The results illustrated that the carbon emissions tax has a positive effect on road network flow equilibrium and carbon emission reduction. This paper will have good reference value and practical significance for the calculation and implementation of urban traffic carbon emissions taxes in China.

## 1 Introduction

With the rapid development of urban economies and motorization, urban transportation has played a key role in urbanization and environmental protection [1, 2]. Traffic congestion, traffic safety and transportation pollution are the three main issues in urban transportation management, and how to effectively solve traffic congestion and transportation pollution in urban areas is a main research issue of transportation management. As one of the important means for energy conservation and emission reduction [3], carbon emissions taxes can effectively reduce air pollution from urban road traffic, improve traffic operation and ease the conflict between transportation supply and demand.

To reduce carbon emissions, researchers have presented many strategies, including the replacement of traditional cars with hybrid-electric vehicles [4] or electric vehicles [5, 6], the integration of land use with transportation management policy [7–10], as well as the carbon emissions tax [11]. Carbon emissions tax can affect trip costs for travelers, which will affect the choices that travelers make, such as the trip destination, departure time, mode choice and routing selection [12, 13]. Within the research field, the influence of the carbon emissions tax on road traffic distribution has attracted a great deal of attention; for example, refer to the research of [14–16].

The emergence of intelligent transportation [17] can achieve real-time monitoring of vehicles’ exhaust emissions and charge consumers based on emission changes. Rilett and Benedek (1994) [18] combined the environmental factors and travel impedance and presented a new traffic distribution method to adapt for environmental protection. In the traditional traffic distribution theory and method, travel impedance considers only the shortest path, which cannot allow for the calculation of minimum energy consumption and environmental pollution emissions. Ahnk (2008) [19] compared the influence on vehicle energy consumption and emission under different route choices.

Most of the research studies are based on comparisons with other forms of charge policies to analyze the effects of the carbon emissions tax on road traffic emission reduction. To reduce road traffic’s carbon emissions, this paper proposed to use a carbon emissions tax to achieve this purpose and analyzed the road network traffic distribution before and after the imposition of the carbon emissions tax. First, a mesoscopic model for calculating carbon emissions tax was established, which was based on road traffic flow and vehicle speed, as well as on carbon emissions. Referring to existing research results to calibrate the value of time, this paper modified the traveler’s generalized cost function, including the carbon emissions tax, fuel surcharge and travel time cost, which can be used in the travel impedance model under the carbon emissions tax. Second, an analysis method for urban road network traffic flow distribution was proposed, and a joint traffic distribution model was built that considered the interaction relationship between private cars and taxis. Finally, we took the city of Panjin as an example to analyze the road traffic carbon emissions tax’s impact on road network traffic flow and road traffic carbon emissions.

The structure of this paper is as follows: section 2 presents the carbon emissions tax calculation method, section 3 establishes the traffic distribution model under the carbon emissions tax, section 4 presents a case study of Panjin, China, and section 5 offers the conclusion.

## 2 Carbon emissions tax calculation method

The carbon emissions tax can be conducted as a charge to the public, so this paper took traffic carbon emission as its object and analyzed the influence of the carbon emissions tax on road traffic flow, aiming to optimize the road traffic system and emission reduction. This section is based on the carbon balance theory to analyze the relationship between fuel consumption and carbon emission, which was used to establish a traffic carbon emissions tax calculation model that will serve as a parameter in the carbon emissions tax’s effect on road traffic flow.

### 2.1 Relationship between vehicle carbon emission and fuel consumption

Vehicle fuels such as gasoline and diesel are composed of hydrocarbons that, after combustion, will generate a mixture of gases, including CO, HC, CO_2_, H_2_O, and NO.

Based on the chemical reaction theory, a vehicle’s carbon emission factor is positively correlated with fuel consumption. Huang and Peng (2011) [20] proved that a vehicle’s carbon emission is related to fuel consumption, demonstrating the relationship as follows:

$$M_{CO_{2}}=\frac{440n\rho }{12}Q$$ (1)

where *Q* is the fuel consumption rate, *L/km*; $M_{CO_{2}}$ is the carbon dioxide emissions factor, *g/km*; *n* is the fuel-carbon mass ratio, with a gasoline value of 0.855 in China; and *ρ* is the fuel density, *kg /L*, while the gasoline value is 0.739 *kg /L* in China.

The relationship between vehicles’ carbon emissions and fuel consumption can be expressed as

$$P=\alpha G\left(v_{s}\right)$$ (2)

where *P* is carbon emissions (g); *G*(*v*_*s*_) is vehicles’ fuel consumption function, (*L*); and *α* is the fuel conversion parameter with the carbon, which is 2.35125 *kg/L*.

### 2.2 Relationship between urban road traffic velocity and traffic volume

According to the classical Greenshields traffic flow model, the relationship between vehicles’ velocity and density ([21]) can be expressed as follows:

$$v_{s}=v_{f}(1-\frac{k}{k_{j}})$$ (3)

where *v*_*s*_ is the traveling velocity (km/h); *v*_*f*_ is the free flow speed (km/h); *k* is the traffic density (*veh/km*); and *k*_*j*_ is the congestion density of traffic (*veh/km*).

However, traffic flow does not strictly follow the original model, especially in China. Chen (2004) [22] combined the Greenshields model with Greenberg models and proposed a new and more fitting formula, as showing in (4):

$$\left\{ \begin{array}{l} v_{s}=\frac{v_{f}}{1+{\left(q/C\right)}^{\psi }}\\ \psi =2.076+2.870{\left(q/C\right)}^{3} \end{array}\right.$$ (4)

where *ψ* is the related parameter; *q*/*C* is the ratio of traffic volume and road capacity; and *v*_*s*_ and *v*_*f*_ are the same as in formula (3).

The verified R square was 0.8687 ([22]). This model can show a better simulation of urban traffic flow in China. Here, the formula can be expressed as *g*(*v*_*f*_,*q*/*C*).

### 2.3 Relationship between vehicle fuel consumption and operation status

The carbon emission efficiency function can be expressed as

$$H=h\left(v_{s}\right)$$ (5)

After extending the above relationship and testing different types of vehicles, we can obtain the fitting relationship between speed and fuel consumption. Then, multiplying by the vehicle’s traveling distance, we can estimate the vehicle’s fuel consumption at a certain speed for a certain distance. The formula can be expressed as

$$G=l\chi h[g\left(v_{f},q/C\right)]$$ (6)

where *χ* is the correction coefficient of a vehicle’s operating condition during peak hours (non-peak hour), and *l* is the vehicle’s driving distance (km).

**Fig 1:**
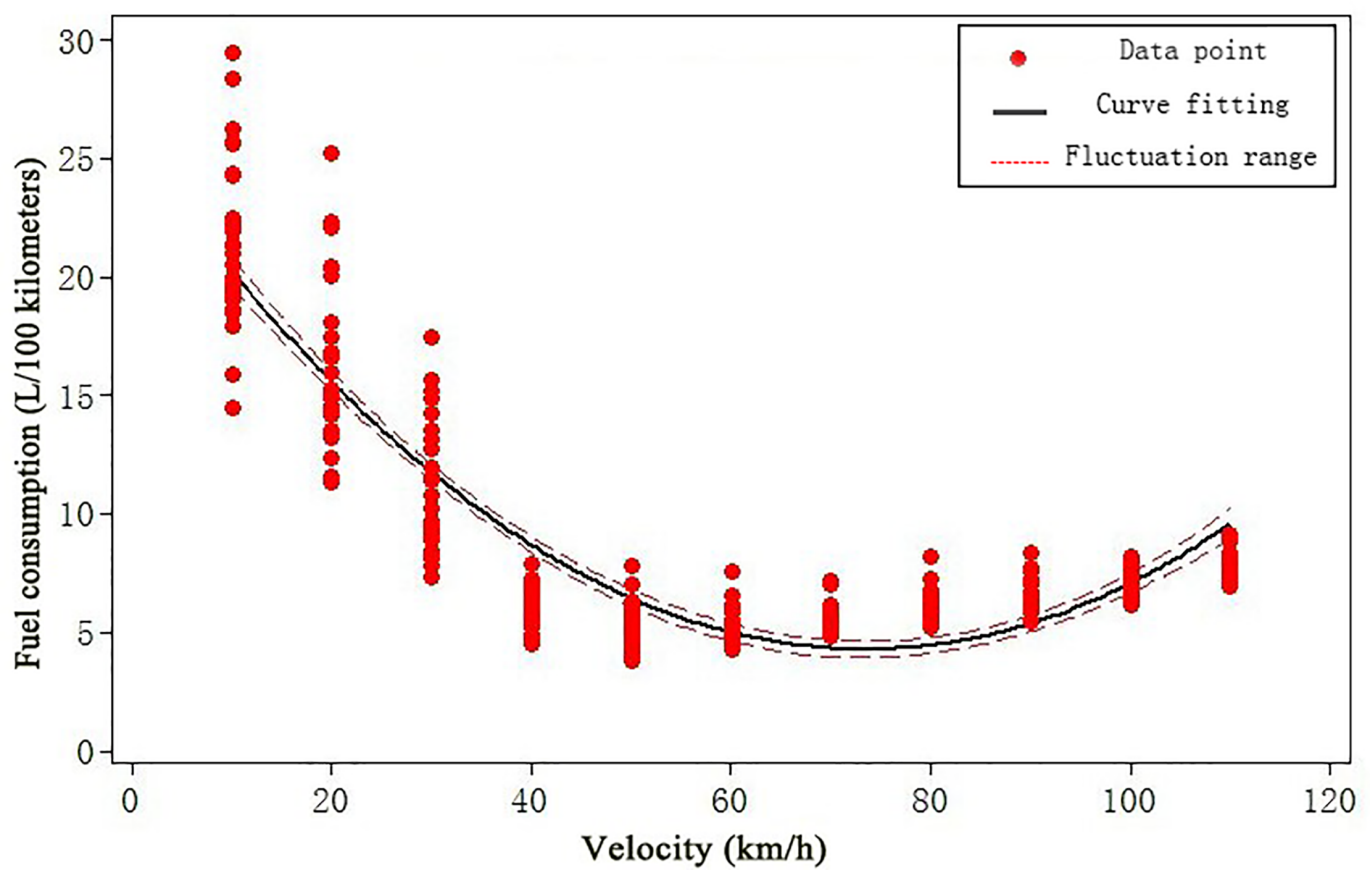
The relationship between fuel consumption and velocity of passenger cars.

**Fig 2:**
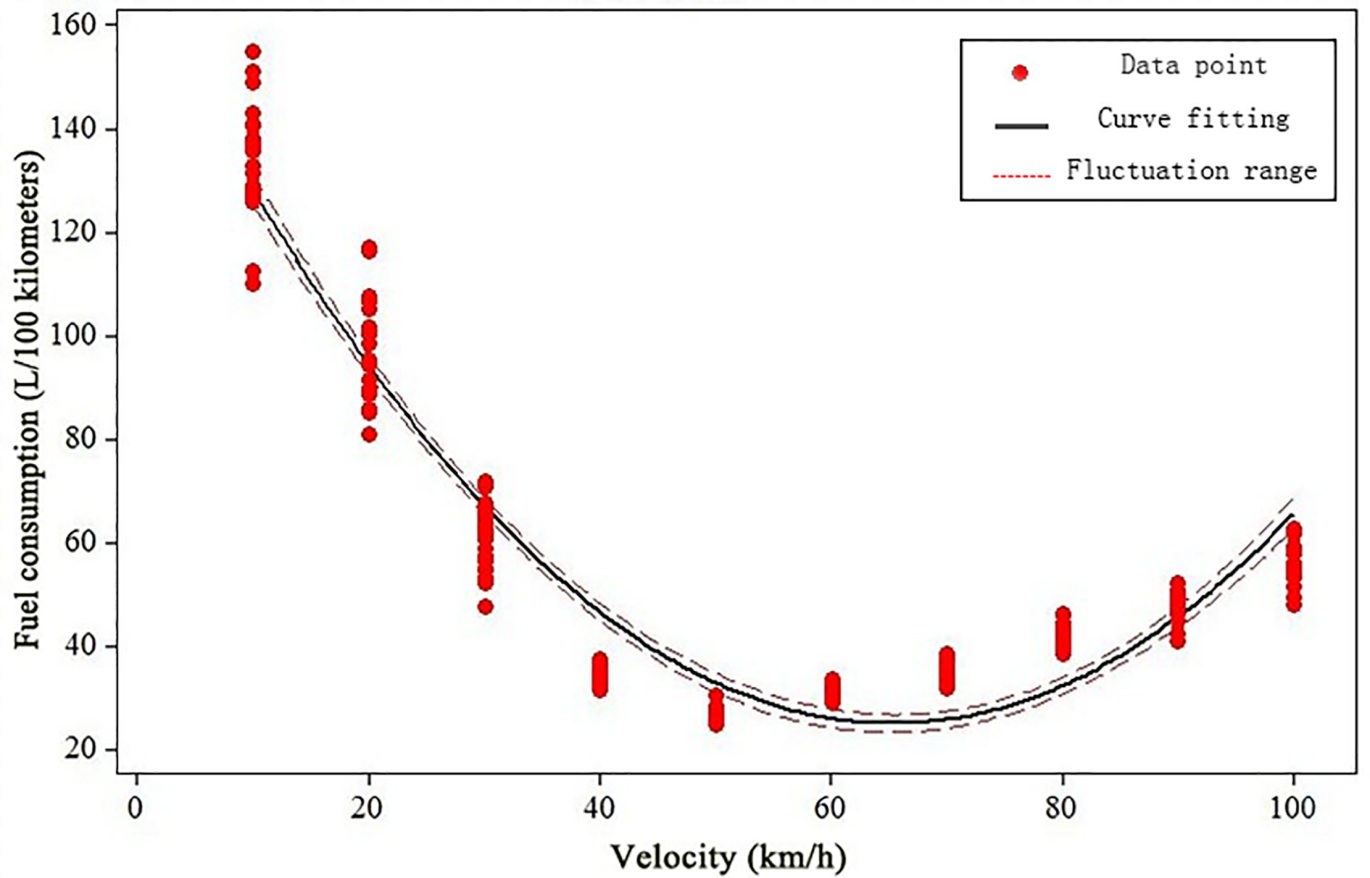
The relationship between fuel consumption and velocity of buses.

Based on survey reports on the fuel consumption and velocity data for different passenger cars and buses, we adopted the Minitab 15 (developed by Minitab Inc.) to fit the relationship between fuel consumption and velocity, and the results of curve fitting for passenger car and bus are given in Fig 1 and Fig 2, respectively.

Thus, the curve fitting of passenger cars’ fuel consumption and velocity can be expressed as

$$H=h\left(v_{s}\right)=0.003968v_{s}^{2}-0.5826v_{s}+25.66\;\left(R^{2}=0.84\right)$$ (7)

The curve fitting of buses’ fuel consumption and velocity can be expressed as

$$H=h\left(v_{s}\right)=0.033732v_{s}^{2}-4.40442v_{s}+168.846\;\left(R^{2}=0.92\right)$$ (8)

### 2.4 Quantification model of vehicles’ carbon emission

Based on the theory of carbon balance and the previous analysis, we can establish the relationship between vehicles’ fuel consumption and carbon emissions based on formulas (2), (5) and (6) and can obtain the vehicle carbon emission model:

$$P=\alpha l\chi h\left(v_{s}\right)$$ (9)

The carbon emission model considers the morning and evening peak-hour characteristics of urban road traffic, as well as the influence of operating speed on fuel consumption, which reflects the reality of the vehicle’s fuel consumption and carbon emissions.

## 3 Traffic distribution model establishments under the carbon emissions tax

### 3.1 The generalized cost function with the consideration of the carbon emissions tax

The general used BPR function can be expressed as

$$t_{a}=t_{0}\left[1+\alpha {\left(\frac{q_{a}}{C_{a}}\right)}^{\beta }\right]$$ (10)

where *α* and *β* are the parameters, with chosen values of 0.15 and 4, respectively; *q*_*a*_ is the traffic flow of the road section *a* (*pcu/h/ln*); *C*_*a*_ is the capacity of the road section *a* (*pcu/h/ln*); *t*_0_ is the free-flow traveling time on road section *a* (s); and *t*_*a*_ is the actual traveling time on road section *a* (s).

This paper mainly focused on motor vehicles, including private cars, taxis and buses. Here, private car and taxi vehicles can be classified as passenger cars, while buses can be classified as another category.

The expense cost function of the traveler comprises the fuel cost, tax cost and other costs. This formula can be expressed as

$$F_{i}=\eta \left(1+\mu \right)Y_{1i}+\xi Y_{2i}$$ (11)

where *F*_*i*_ is the traveler’s expense cost on road section *i*, and *μ* is the carbon emission rate and represents the ratio of carbon emission price and fuel price. *Y*_1*i*_ is the fuel price without a carbon emissions tax on road section *i*; *Y*_2*i*_ is other charges on road section *i*, including the road toll charge, congestion pricing, and so on. *η* and *ξ* are the weight coefficients. In addition,

η+ξ=1 (12)

To acquire the weight coefficients, this paper investigated the opinion of professional experts (including 20 professionals from traffic management agencies) and then adopted an expert scoring method to acquire the values. The value of *ξ* is 0.1, and the value of *η* is 0.9.

The generalized traveling cost function was composed of expense cost and time cost, which can be expressed by the following formula:

$$M_{i}=T_{i}\gamma +F_{i}$$ (13)

where *γ* is the value of time; *T*_*i*_ is the traveling time on road section *i*; and *M*_*i*_ is the traveler’s generalized traveling cost on road section *i*.

Combining formula (13) with formulas (6), (10), and (11), we can obtain the generalized traveling cost with the consideration of the carbon emissions tax and present it in formula (14):

$$M_{i}=\gamma \frac{L_{i}}{g\left(v_{f},q_{i}/C_{i}\right)}+\eta \left(1+\mu \right)L_{i}h\left[g\left(v_{f},q_{i}/C_{i}\right)\right]+\xi Y_{2i}$$ (14)

### 3.2 Estimation the value of time

The valuation of time will depend on the traveler’s condition, such as trip purpose, time urgency, weather conditions and personal income level [23].

Based on a Stated Preferences survey, researchers can obtain the value of time for travelers of different income levels. In Zhang’s (2012) research [24], he obtained the value of travel time based on the traveler’s trip preference. This paper combined the previous research and adopted the value of time for private car, taxi and bus as 15.25 Yuan/h, 14.29 Yuan/h, and 7.51 Yuan/h, respectively.

### 3.3 Traffic flow distribution forecasting method

Here, we present three assumptions of the traffic flow distribution forecasting method.

1. This paper focuses on urban passenger transportation, and the vehicle types include private car, taxi and bus. However, the carbon emissions tax has no effect on bus routing choice, so this paper will first lay out bus routes in the road network and then discuss the competitive relationship between private cars and taxis.
2. Here, we do not consider travelers who choose to walk or take the subway, instead focusing only on the travel mobility modes in the urban road network.
3. This paper considers only the influence on the traffic flow and ignores the influence of road surface and roadside clearance.

The competitive relationship between private cars and taxis can be described as follows:

1. In the multi-mode traffic flow, the increase or decrease of one private car vehicle not only affects the impedance of that private car but also affects the taxi mode; in the same way, the increase or decrease of one taxi will have the same effect, and this effect is symmetrical.
2. The chain affecting the flow change by one type of vehicle will have a bigger influence on itself than on other types of vehicles. Here, we assume that the mutual influence of taxis will be larger than private cars’ influence on taxis; otherwise, the mutual influence of private cars will be larger than the taxis’ influence on private cars.

Based on the traffic flow assumption, we will first lay out the bus flow in the urban road network, so the basic traffic flow will be

$$q_{0i}=\varpi \sum_{j=1}^{n}\frac{60}{d_{j}}$$ (15)

where *q*_0*i*_ is the bus flow on road section *i pcu/h*; *ϖ* is the passenger car equivalence between bus and private car, with a value here of 2.0; and *d*_*j*_ is the departure interval of the bus *j*, *min/pcu*.

The impedance functions of taxis and private cars can be expressed as follows:

$$t_{i}=\gamma \frac{L_{i}}{v_{fi}}\left[1+\alpha {\left(\frac{q_{0i}+w_{i}+{\hat{w}}_{i}}{C_{i}}\right)}^{\beta }\right]+\left(1+\mu \right)L_{i}\chi h\left(v_{s}\right)$$ (16)

$${\hat{t}}_{i}=\hat{\gamma }\frac{L_{i}}{v_{fi}}\left[1+\alpha {\left(\frac{q_{0i}+w_{i}+{\hat{w}}_{i}}{C_{i}}\right)}^{\beta }\right]+\left(1+\mu \right)L_{i}\chi h\left(v_{s}\right)$$ (17)

where *γ* is the value of time for private cars; $\hat{\gamma }$ is the value of time for taxis; *w*_*i*_ is the traffic flow of private cars on road section *i*; and ${\hat{w}}_{i}$ is the traffic flow of taxis on road section *i*.

In the routing choice process, travelers will make decisions based on the above impedance, and each traveler’s random choice probability can be expressed as

$$P_{j}=\frac{e^{-a_{1}t_{j}}}{\sum_{p}e^{-a_{1}t_{i}}}$$ (18)

where *a*_1_ is the calculating parameter.

The combination model of road traffic assignment is a linear programming model, and the objective function is the minimum of the total road network’s generalized traveling cost. Based on the research of [25], this paper substituted the carbon emissions tax and different modes’ time values into the traffic flow model, which combined the mode split and flow assignment. The model can be expressed as follows:

$$\begin{array}{l} \min Z\left(w,\hat{w},q_{rs},{\hat{q}}_{rs},Q_{rs}\right)=\sum_{a}\frac{1}{2}\left\{\int_{0}^{w_{i}}\left[t_{i}\left(\omega ,{\hat{w}}_{i}\right)+t_{i}\left(\omega ,0\right)\right]d\omega +\int_{0}^{{\hat{w}}_{i}}\left[{\hat{t}}_{i}\left(w_{i},\upsilon \right)+{\hat{t}}_{i}\left(0,\upsilon \right)\right]d\upsilon \right\}\\ +\frac{1}{a_{1}}\sum_{r,s,p}\left(\int_{0}^{f_{p}^{rs}}\ln\omega d\omega +\int_{0}^{{\hat{f}}_{p}^{rs}}\ln\omega d\omega \right)+\left(\frac{1}{a_{2}}-\frac{1}{a_{1}}\right)\sum_{r,s,p}\left(\int_{0}^{q_{rs}}\ln\omega d\omega +\int_{0}^{{\hat{q}}_{rs}}\ln\omega d\omega \right)\\ +\sum_{r,s}\left(Q_{rs}\ln Q_{rs}-Q_{rs}\right) \end{array}$$ (19)

Subject to:

$$\sum_{p}f_{p}^{rs}=q_{rs},\forall r,s;$$

$$\sum_{p}{\hat{f}}_{p}^{rs}={\hat{q}}_{rs},\forall r,s;$$

$$\sum_{s}Q_{rs}=R_{r},\forall r,s;$$

$$\sum_{s}Q_{rs}=S_{s},\forall r,s;$$

$$w_{i}=\sum_{r,s,p}f_{p}^{rs}\delta_{i,p}^{rs},\forall p;$$

$${\hat{w}}_{i}=\sum_{r,s,p}{\hat{f}}_{p}^{rs}\delta_{i,p}^{rs},\forall p;$$

$$q_{rs}+{\hat{q}}_{rs}=Q_{rs},\forall r,s;$$

$$f_{p}^{rs}\geq 0,\;{\hat{f}}_{p}^{rs}\geq 0,\;x_{a}\geq 0,\;{\hat{x}}_{a}\geq 0,\;q_{rs}\geq 0,\;{\hat{q}}_{rs}\geq 0,\;Q_{rs}\geq 0$$

where *Q*_*rs*_ is the traffic flow between OD pairs (r, s); $f_{p}^{rs}$ is the private car’s flow on the path *p* between OD pairs (r, s); ${\hat{f}}_{p}^{rs}$ is the taxi’s flow on the path *p* between OD pairs (r, s); *q*_*rs*_ is the private car’s flow between OD pairs (r, s); ${\hat{q}}_{rs}$ is the taxi’s flow between OD pairs (r, s); and $\delta_{i,p}^{rs}$ is the correlation coefficient, such that if road section *i* in the routing *p*, the value will be 1; otherwise, it will be 0. *S*_*s*_ is the origin traffic production. *R*_*r*_ is the destination traffic attraction.

The objective of function (19) is a strictly convex, and the constraint conditions of this model are convex set, the combination model is convergent. This combination model can be proven equivalent with the integrated computation for mode split and mode’s effect [25], which will be proved by the Lagrange function to transfer this combination model’s first-order condition.

### 3.4 Solution method

The combination model’s solution method can be expressed as follows:

Step 1: Determine the bus route and departure interval, as well as the basic bus traffic flow *q*_0*i*_.

Step 2: Under the given destination traffic attraction *R*_*r*_ and origin traffic production *S*_*s*_, set the iterative initial value, which includes the *Q*_*rs*_, $f_{p}^{rs}$, ${\hat{f}}_{p}^{rs}$, $q_{p}^{rs}$, and ${\hat{q}}_{p}^{rs}$, then define an integer variable *k*; here, the initial value is 1.

Step 3: According to the value of *Q*_*rs*_, $f_{p}^{rs}$, ${\hat{f}}_{p}^{rs}$, $q_{p}^{rs}$, and ${\hat{q}}_{p}^{rs}$, calculate the generalized cost.

Step 4: Based on the impedance of private car and taxi (formula 16 and 17) and the value of *R*_*r*_ and *S*_*s*_, use the Furness traffic distribution forecasting method to obtain the different traffic zones’ traffic exchange volume $q_{p}^{rs'}$ and ${\hat{q}}_{p}^{rs'}$.

Step 5: Calculate the value of $f_{p\;k}^{rs\;'}$ and ${\hat{f}}_{p\;k}^{rs\;'}$, and statistics for the road section traffic volume *Q*_*rs k*_', *w*_*ik*_ and ${\hat{w}}_{i\;k}$.

Step 6: Adopt routing choice logit model to obtain the new road section volume $f_{p\;k+1}^{rs}$, ${\hat{f}}_{p\;k+1}^{rs}$, $q_{p\;k+1}^{rs}$ and ${\hat{q}}_{p\;k+1}^{rs}$, with the following iterative formulas:

$$e=\frac{1}{k+1}$$ (20)

$$f_{p\;k+1}^{rs}=f_{p\;k}^{rs}+e\left(f_{p\;k}^{rs\;'}-f_{p\;k}^{rs}\right)$$ (21)

$${\hat{f}}_{p\;k+1}^{rs}={\hat{f}}_{p\;k}^{rs}+e\left({\hat{f}}_{p\;k}^{rs\;'}-{\hat{f}}_{p\;k}^{rs}\right)$$ (22)

$$q_{p\;k+1}^{rs}=q_{p\;k}^{rs}+e\left(q_{p\;k}^{rs\;'}-q_{p\;k}^{rs}\right)$$ (23)

$${\hat{q}}_{p\;k+1}^{rs}={\hat{q}}_{p\;k}^{rs}+e\left({\hat{q}}_{p\;k}^{rs\;'}-{\hat{q}}_{p\;k}^{rs}\right)$$ (24)

Qrsk+1=Qrsk+e(Qrsk'−Qrsk) (25)

k=k+1 (26)

Step 7: Verify convergence:

$$\left|f_{p\;k+1}^{rs}-f_{p\;k}^{rs}\right|+\left|{\hat{f}}_{p\;k+1}^{rs}-{\hat{f}}_{p\;k}^{rs}\right|+\left|q_{p\;k+1}^{rs}-q_{p\;k}^{rs}\right|+\left|{\hat{q}}_{p\;k+1}^{rs}-{\hat{q}}_{p\;k}^{rs}\right|+\left|Q_{rs\;k+1}-Q_{rs\;k}\right|\leq \varepsilon$$ (27)

where *ε* is the convergence precision value. If the variables meet, stop the iteration; otherwise, go back to step 3.

## 4 Case study of Panjin, China

### 4.1 Parameters setting

**Fig 3:**
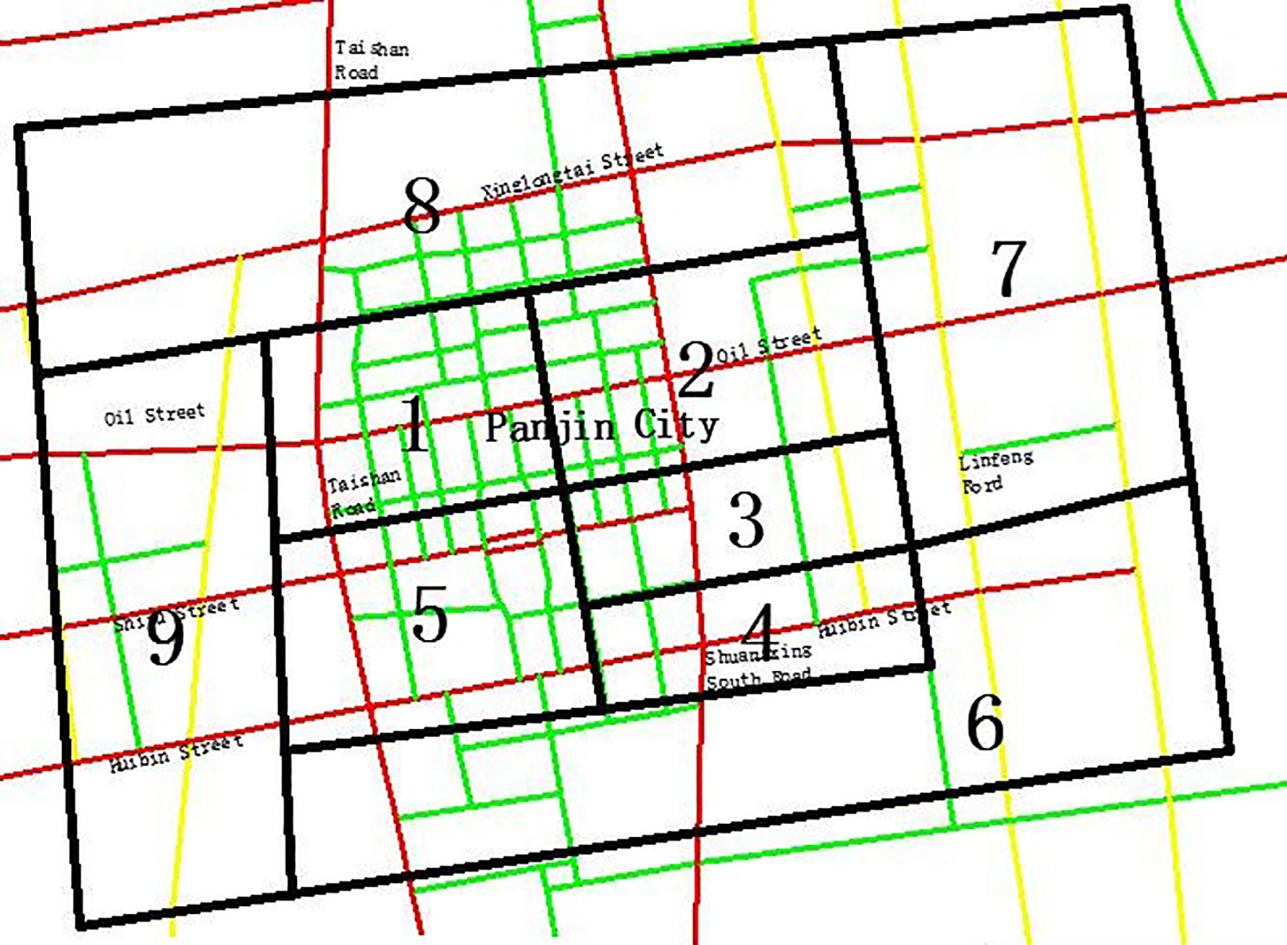
Research area of Panjin city.

The case study area chosen as the basic influence area for the carbon emissions tax was one part of a road network in Panjin carbon emissions tax, including 9 traffic zones and 30 road sections. Table 1 shows the trip productions and attractions in the research area, and the detailed information on the research area is shown in Fig 3. According to the intersection and road section traffic volume, we can obtain the main road section’s volume information, which is shown in S1 Table.

**Table 1:** Trip productions and attractions in the research area.

Traffic zone	1	2	3	4	5	6	7	8	9
Production (trips per day)	6,102	9,292	8,209	6,013	11,145	40,438	34,904	30,368	31,646
Attractions (trips per day)	21,840	1,5805	7,583	7,378	26,575	22,981	19,068	30,109	26,778

### 4.2 Carbon emissions tax pricing

Based on the vehicle population and fuel consumption of Panjin from 2005 to 2011, which is from Panjin Statistical Yearbook from 2006 to 2012, this paper derived the carbon emission’s shadow price as the standard of the carbon emissions tax. Table 2 presents the statistics for the vehicle population of Panjin.

**Table 2:** Vehicle population of Panjin from 2005 to 2011 * (vehicle).

Year	2005	2006	2007	2008	2009	2010	2011
Truck	13,359	14,481	15,745	17,181	22,176	26,032	29,289
Passenger car	35,388	43,248	52,380	63,925	79,307	97,447	119,843

*Data source are from 2006 to 2012 Panjin Statistical Yearbooks.

Table 3 gives the statistics for vehicle fuel consumption in Panjin.

**Table 3:** Vehicle fuel consumption in Panjin from 2005 to 2011 * (thousands of tons).

Year	2005	2006	2007	2008	2009	2010	2011
Gasoline	36	19	24	249	34.7	20.5	41
Diesel	135	181	193	209	139	131.8	174

*Data source are from 2006 to 2012 Panjin Statistical Yearbooks.

Meanwhile, based on the relationship between fuel quality and volume transfer, we can calculate Panjin’s carbon emission from 2005 to 2011. Here, we adopted the density of gasoline value as 0.739 g/ml, as well as diesel density as 0.86 g/ml.

According to the requirements of the shadow model, with the combination of the transportation industry’s input-output parameters, we can calculate the shadow price of carbon emission for different years. We calculated the explanatory variable of the directional distance function. The generalized maximum likelihood method was used to estimate the effective frontier. Table 4 presents the carbon emission shadow prices. As shown in Table 4, the shadow price of carbon emission is not invariable but is correlated with the input and output of transportation industry.

**Table 4:** Carbon emission prices in different years.

Year	2005	2006	2007	2008	2009	2010	2011
Carbon emission shadow price (¥ per ton)	344.6	401.0	404.9	528.8	434.0	363.1	552.6

This paper took the carbon emission shadow price as the standard for the carbon emissions tax and took the average shadow price from 2009 to 2011 as the carbon emissions tax in 2012. According to the transfer formula, we obtained the gasoline and diesel price increase as 1.2 Yuan/L and 1.3 Yuan/L, respectively, while the current gasoline price is 7.3 Yuan/L; thus, we obtain a carbon emissions tax rate of 0.164.

### 4.3 Traffic flow distribution forecasting results under the carbon emissions tax

Here, we chose the convergence precision *ε* = 1%; the average number of passengers for buses is 23 persons per vehicle, taxis are 2.1 persons per vehicle, and private cars are 1.8 persons per vehicle. According to the above iterative calculation method, we obtain the road network flow and V/C before and after the implementation of the carbon emissions tax in Panjin.

According to Eq (15), we can get the bus traffic volume in the research area, as shown in S2 Table. Based on the iteration method of section 3.3, we can get the final results of the traffic volume and saturation degree (v/c ratio) for each road section, which are shown in S3 Table.

Before the implementation of the carbon emissions tax, the V/C variance is 0.01285, while after the implementation of the carbon emissions tax, the variance is 0.009706. The traffic flow’s balance influence is more noticeable, such as the road section of Linfeng Road from Xinglongtai street to Oil street, the traffic volume dropped from 3018 pcu/h to 2752 pcu/h, and the v/c ratio is changed from 0.629 to 0.573

### 4.4 Carbon emission within the road network

According to formula (9), we can obtain the total carbon emission of the road network, and the results are shown in Table 5. As shown in Table 5, the total carbon emission in the road network was reduced by 0.66%. Under the road congestion and flow imbalance road sections, the effect will be more remarkable.

**Table 5:** Carbon emission in the road network (kg/h).

	Before carbon emissions tax	After carbon emissions tax	Change
Carbon emission	9,347.5	9,286.3	-61.2

From the above analysis, it is clear that the carbon emissions tax has a positive influence on road network traffic flow balance and increases road-use efficiency. Furthermore, from the road network carbon emissions, we find that after the implementation of the carbon emissions tax, the total carbon emissions will reduce, reflecting the carbon emissions tax’s effect. Meanwhile, Barnett and Knibbs (2014) [26] found that higher diesel prices were connected with short-term reductions in carbon monoxide and would have a beneficial impact on public health.

## 5 Conclusions

Aiming to analyze the carbon emissions tax’s effect on road traffic and emission reduction, this paper established a mesoscopic model for calculating the carbon emissions tax that is based on the relationship between urban road traffic flow and speed. Then, we modified the traveler’s generalized cost function and established the road network traffic flow distribution forecasting method under the carbon emissions tax policy, which considered the interaction relationship between private cars and taxis. A case study of Panjin was used to verify the proposed model’s effectiveness and proved the influence of carbon emissions tax on traffic flow balance and emission reduction.

Combining the statistical data from Panjin city and the traffic carbon emission model, we predicted the carbon emission from road traffic and used the shadow price model to calculate the carbon emission price. Based on the road network traffic distribution, we obtained the traffic flow re-distribution after the implementation of the carbon emissions tax and found that the carbon emissions tax has a positive effect on road network flow equilibrium and carbon emission reduction.

In the future, more efforts will be directed to the carbon emissions tax’s implementation scope and the combination of congestion pricing and carbon emission field [27], aiming to achieve more effective carbon emission reduction results; meanwhile, with the development of electric vehicles, the carbon emissions for the total life cycle should be further discussed.

## Supporting information

S1 Table The information of road sections in research area. (DOCX)

S2 Table Bus traffic volume in the research area (pcu/h). (DOCX)

S3 Table Final results of the research road network before and after the congestion pricing. (DOCX)
