# Supplementary material for: Carbon emissions tax policy of urban road traffic and its application in Panjin, China
Source: PLoS One. 2018 May 8;13(5):e0196762. doi: 10.1371/journal.pone.0196762 (PMC5940227; doi:10.1371/journal.pone.0196762)
Supplement: S1 Table — (DOCX) [file pone.0196762.s001.docx]

S1Table . The information of road sections in research area

| Road | Section | Lane number  (two way) | | Capacity (pcu/h) | Length (km) |
| --- | --- | --- | --- | --- | --- |
| Xinglongtai Street | Xingyou Branch- Taishan road | 6 lanes | | 6800 | 0.32 |
|  | Taishan road- Shuangxing South Road | 6 lanes | | 6800 | 1.21 |
|  | Shuangxing South Road- Yingbin Road | 6 lanes | | 6800 | 0.52 |
|  | Yingbin Road- Linfeng Road | 6 lanes | | 6800 | 0.41 |
|  | Linfeng Road-Xiangdao Road | 4 lanes | | 4800 | 0.55 |
| Oil Street | Xingyou Branch- Taishan road | | 8 lanes | 8900 | 0.15 |
|  | Taishan road- Shuangxing South Road | | 10 lanes | 10600 | 0.33 |
|  | Shuangxing South Road- Yingbin Road | | 8 lanes | 8900 | 1.22 |
|  | Yingbin Road- Linfeng Road | | 6 lanes | 6800 | 0.54 |
|  | Linfeng Road- Xiangdao Road | | 6 lanes | 6800 | 0.46 |
| City Hall Street | Xingyou Branch- Taishan road | | 6 lanes (Separated) | 7000 | 0.46 |
|  | Taishan road- Shuangxing South Road | | 6 lanes (Separated) | 7001 | 1.21 |
| Huibin Street | Xingyou Branch- Taishan road | | 4 lanes | 4800 | 0.61 |
|  | Taishan road- Shuangxing South Road | | 6 lanes | 6800 | 1.11 |
|  | Shuangxing South Road- Yingbin Road | | 6 lanes | 6800 | 0.55 |
|  | Yingbin Road- Linfeng Road | | 6 lanes | 6600 | 0.36 |
|  | Linfeng Road-Xiangdao Road | | 6 lanes | 6600 | 0.27 |
| Xingyou Branch | Xinglongtai Street- Oil Street | | 4 lanes | 4800 | 0.63 |
|  | Oil Street- City Hall Street | | 4 lanes | 4800 | 0.52 |
|  | City Hall Street- Huibin Street | | 4 lanes | 4800 | 0.47 |
| Taishan Road | Xinglongtai Street- Oil Street | | 8 lanes | 8900 | 0.68 |
|  | Oil Street- City Hall Street | | 10 lanes | 10600 | 0.45 |
|  | City Hall Street- Huibin Street | | 8 lanes | 8600 | 0.44 |
| Shuangxing South Road | Xinglongtai Street- Oil Street | | 10 lanes | 10600 | 0.71 |
|  | Oil Street- City Hall Street | | 10 lanes | 10600 | 0.46 |
|  | City Hall Street- Huibin Street | | 10 lanes | 10600 | 0.47 |
| Yingbin Road | Xinglongtai Street- Oil Street | | 6 lanes | 6600 | 0.64 |
|  | City Hall Street- Huibin Street | | 4 lanes | 4800 | 0.71 |
| Linfeng Road | Xinglongtai Street- Oil Street | | 4 lanes | 4800 | 0.65 |
|  | City Hall Street- Huibin Street | | 4 lanes | 6800 | 0.63 |
| Xiangdao Road | Xinglongtai Street- Oil Street | | 4 lanes | 4800 | 0.61 |
|  | City Hall Street- Huibin Street | | 4 lanes | 4800 | 0.61 |
