# Supplementary material for: Carbon emissions tax policy of urban road traffic and its application in Panjin, China
Source: PLoS One. 2018 May 8;13(5):e0196762. doi: 10.1371/journal.pone.0196762 (PMC5940227; doi:10.1371/journal.pone.0196762)
Supplement: S2 Table — (DOCX) [file pone.0196762.s002.docx]

S2 Table . Bus traffic volume in the research area (pcu/h)

| Road | Section | Bus traffic volume | Road | Section | Bus traffic volume |
| --- | --- | --- | --- | --- | --- |
| Xinglongtai Street | Xingyou Branch- Taishan road | 27 | Huibin Street | Xingyou Branch- Taishan road | 0 |
|  | Taishan road- Shuangxing South Road | 63 |  | Taishan road- Shuangxing South Road | 24 |
|  | Shuangxing South Road- Yingbin Road | 103 |  | Shuangxing South Road- Yingbin Road | 36 |
|  | Yingbin Road- Linfeng Road | 88 |  | Yingbin Road- Linfeng Road | 36 |
|  | Linfeng Road-Xiangdao Road | 51 |  | Linfeng Road-Xiangdao Road | 8 |
| Oil Street | Xingyou Branch- Taishan road | 0 | Xingyou Branch | Xinglongtai Street- Oil Street | 0 |
|  | Taishan road- Shuangxing South Road | 64 |  | Oil Street- City Hall Street | 0 |
|  | Shuangxing South Road- Yingbin Road | 40 |  | City Hall Street- Huibin Street | 0 |
|  | Yingbin Road- Linfeng Road | 40 | Taishan Road | Xinglongtai Street- Oil Street | 44 |
|  | Linfeng Road- Xiangdao Road | 68 |  | Oil Street- City Hall Street | 72 |
| City Hall Street | Xingyou Branch- Taishan road | 12 |  | City Hall Street- Huibin Street | 36 |
|  | Taishan road- Shuangxing South Road | 20 | Shuangxing South Road | Xinglongtai Street- Oil Street | 36 |
| Linfeng Road | Xinglongtai Street- Oil Street | 47 |  | Oil Street- City Hall Street | 20 |
|  | City Hall Street- Huibin Street | 12 |  | City Hall Street- Huibin Street | 12 |
| Xiangdao Road | Xinglongtai Street- Oil Street | 52 | Yingbin Road | Xinglongtai Street- Oil Street | 55 |
|  | City Hall Street- Huibin Street | 8 |  | City Hall Street- Huibin Street | 8 |
