# Supplementary material for: Carbon emissions tax policy of urban road traffic and its application in Panjin, China
Source: PLoS One. 2018 May 8;13(5):e0196762. doi: 10.1371/journal.pone.0196762 (PMC5940227; doi:10.1371/journal.pone.0196762)
Supplement: S3 Table — (DOCX) [file pone.0196762.s003.docx]

S3 Table. Final results of the research road network before and after the congestion pricing

| Road | Section | Before Carbon Emission Tax Policy | | After Carbon Emission Tax Policy | |
| --- | --- | --- | --- | --- | --- |
|  |  | Traffic volume | v/c ratio | Traffic volume | v/c ratio |
| Xinglongtai Street | Xingyou Branch- Taishan road | 3186 | 0.469 | 3213 | 0.473 |
|  | Taishan road- Shuangxing South Road | 4022 | 0.591 | 3954 | 0.581 |
|  | Shuangxing South Road- Yingbin Road | 3201 | 0.471 | 3230 | 0.475 |
|  | Yingbin Road- Linfeng Road | 2518 | 0.37 | 2626 | 0.386 |
|  | Linfeng Road-Xiangdao Road | 3032 | 0.632 | 2753 | 0.574 |
| Oil Street | Xingyou Branch- Taishan road | 2779 | 0.312 | 2779 | 0.312 |
|  | Taishan road- Shuangxing South Road | 4195 | 0.396 | 3982 | 0.376 |
|  | Shuangxing South Road- Yingbin Road | 3782 | 0.425 | 3827 | 0.43 |
|  | Yingbin Road- Linfeng Road | 2613 | 0.384 | 2754 | 0.405 |
|  | Linfeng Road- Xiangdao Road | 1982 | 0.291 | 1849 | 0.272 |
| City Hall Street | Xingyou Branch- Taishan road | 2471 | 0.353 | 2735 | 0.391 |
|  | Taishan road- Shuangxing South Road | 2813 | 0.402 | 3041 | 0.434 |
| Huibin Street | Xingyou Branch- Taishan road | 3087 | 0.643 | 2679 | 0.558 |
|  | Taishan road- Shuangxing South Road | 2876 | 0.423 | 2753 | 0.405 |
|  | Shuangxing South Road- Yingbin Road | 3130 | 0.46 | 2892 | 0.425 |
|  | Yingbin Road- Linfeng Road | 1903 | 0.288 | 1974 | 0.299 |
|  | Linfeng Road-Xiangdao Road | 1709 | 0.259 | 1832 | 0.278 |
| Xingyou Branch | Xinglongtai Street- Oil Street | 1825 | 0.38 | 1975 | 0.411 |
|  | Oil Street- City Hall Street | 1623 | 0.338 | 1745 | 0.364 |
|  | City Hall Street- Huibin Street | 1572 | 0.328 | 1613 | 0.336 |
| Taishan Road | Xinglongtai Street- Oil Street | 4127 | 0.464 | 3874 | 0.435 |
|  | Oil Street- City Hall Street | 3449 | 0.325 | 3479 | 0.328 |
|  | City Hall Street- Huibin Street | 3671 | 0.427 | 3563 | 0.414 |
| Shuangxing South Road | Xinglongtai Street- Oil Street | 4607 | 0.435 | 4639 | 0.438 |
|  | Oil Street- City Hall Street | 4402 | 0.415 | 4577 | 0.432 |
|  | City Hall Street- Huibin Street | 2509 | 0.237 | 2582 | 0.244 |
| Yingbin Road | Xinglongtai Street- Oil Street | 1544 | 0.234 | 1793 | 0.272 |
|  | City Hall Street- Huibin Street | 1159 | 0.241 | 1325 | 0.276 |
| Linfeng Road | Xinglongtai Street- Oil Street | 3018 | 0.629 | 2752 | 0.573 |
|  | City Hall Street- Huibin Street | 2632 | 0.387 | 2647 | 0.389 |
